# The RED domain of Paired is specifically required for *Drosophila* accessory gland maturation

**DOI:** 10.1098/rsob.140179

**Published:** 2015-02-18

**Authors:** Li Li, Ping Li, Lei Xue

**Affiliations:** Institute of Intervention Vessel, Shanghai 10th People's Hospital, Shanghai Key Laboratory of Signaling and Disease Research, School of Life Science and Technology, Tongji University, 1239 Siping Road, Shanghai 200092, People's Republic of China

**Keywords:** accessory gland, post-mating response, Paired, RED domain, *Drosophila*

## Abstract

The evolutionarily conserved paired domain consists of the N-terminal PAI and the C-terminal RED domains, each containing a helix–turn–helix motif capable of binding DNA. Despite its conserved sequence, the physiological functions of the RED domain remain elusive. Here, we constructed a *prd* transgene expressing a truncated Paired (Prd) protein without the RED domain, and examined its rescue ability in *prd* mutants. We found that the RED domain is specifically required for the expression of Acp26Aa and sex peptide in male accessory glands, and the induction of female post-mating response. Our data thus identified an important physiological function for the evolutionarily conserved RED domain.

## 2. Introduction

The *Drosophila gene paired* (*prd*) belongs to the pair-rule gene family that exhibits a pair-rule expression pattern and participates in the determination of anterior–posterior axis during early embryogenesis [1–4]. Together with other pair-rule genes, *prd* regulates the expression of segment-polarity genes, including *wingless* (*wg*), *gooseberry* (*gsb*) and *engrailed* (*en*) [3,5–7]. In addition to the embryonic functions, *prd* is necessary for postembryonic viability, and male fertility through the regulation of accessory gland (AG) development [8–12]. The *Drosophila* male AG is a pair of dead-end tubes composed of a single-cell layer of two kinds of secretory cells: the ‘main cells’ spreading all over the lumen of the glands, and the ‘secondary cells’ restricted only to the distal region of each tube [13]. The AG is a secretory organ that is functionally analogous to the human prostate and seminal vesicle whose secretions (seminal fluid), together with sperm from the testes, play important roles in regulating female post-mating response (PMR) [11,13–17].

*prd* is the founding member of the *Pax* genes, which encode an evolutionarily conserved family of transcription factors with multiple DNA binding motifs and play key roles in animal development [18–21]. All members of the Pax family are defined by the presence of a highly conserved 128-amino-acid paired domain (PD) [22], which was first identified in Paired (Prd) [23]. The crystal structure demonstrates that PD is a bipartite module, which is further divided into the N-terminal PAI and the C-terminal RED subdomains (PAI + RED = PD) [24]. Each subdomain contains a helix–turn–helix (HTH) motif and has the ability to bind to DNA independently [24,25]. In addition to PD, some Pax proteins also contain a paired-type homeodomain (HD). According to the different combinations of these domains, the Pax proteins are classified into five subgroups [26]. Prd is homologous to the mammalian Pax3/Pax7 subgroup containing both a complete PD and an intact HD. It has been reported that both HD and PAI subdomain are simultaneously required within the same molecule to execute the early embryonic pair-rule function of Prd [8], whereas the RED subdomain appears dispensable for these functions either *in vitro* [27] or *in vivo* [8].

To investigate whether the RED subdomain is important for other Prd functions, we introduced a transgene, *prd*-PrdΔPBC, which encodes a truncated Prd protein with a deletion of the RED domain under the control of the complete *cis*-regulatory region, into a *prd* null mutant background. We found that the RED domain is dispensable for most Prd functions, including embryonic segmentation, postembryonic viability, AG development and male fertility, but is specifically required for the expression of Acp26Aa (also called Ovulin) and sex peptide (SP), two seminal fluid components essential for the induction of female PMR. Consequently, *prd* mutant males rescued by *prd*-PrdΔPBC failed to induce increased egg laying and decreased receptivity in wild-type females. Thus, we have characterized a specific function for the RED domain of Prd protein, which has shed light on the evolution of *Pax* genes.

## 3. Results and discussion

### 3.1. The RED domain is dispensable for the embryonic functions of Prd

**Figure 1.**
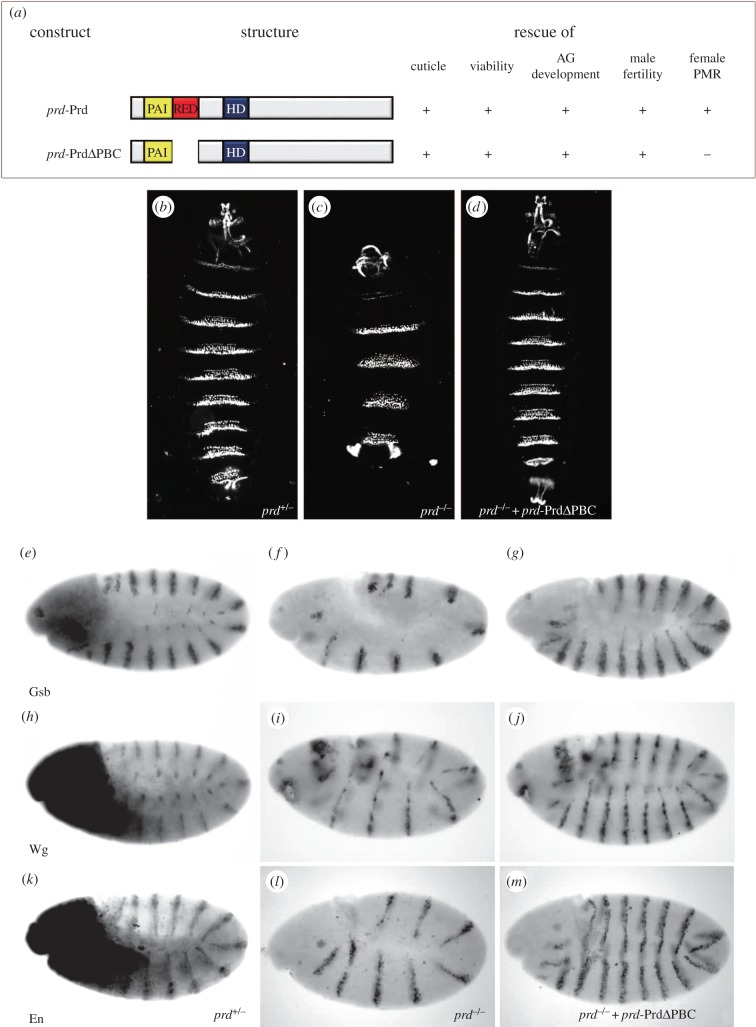
The RED domain is dispensable for the embryonic functions of Prd. (*a*) Schematic of the coding structure of *prd*-Prd and *prd*-PrdΔPBC, and their ability to rescue Prd functions in *Drosophila* development. The rescue ability is scored by + if the transgene is sufficient for rescue or by − if no rescue is obtained. (*b*–*d*) The cuticle of a *prd^+/−^* (*b*), or a *prd^−/−^* (*c*) or a *prd^−/−^; prd*-PrdΔPBC/+ (*d*) embryo is shown under dark-field illumination with anterior up. *prd*-PrdΔPBC is able to rescue the cuticle phenotype in *prd*^−^ (*d*). Expression patterns of Gsb (*e*–*g*), Wg (*h*–*j*) and En (*k*–*m*) in *prd^+/−^* (*e*,*h,k*), *prd^−/−^*embryo carrying no (*f*,*i*,*l*) or one copy of *prd*-PrdΔPBC (*g*,*j,m*) are shown during the extended germ band stage of embryogenesis. Embryos are oriented with their anterior to the left and dorsal side up. Expression of Gsb, Wg and En in *prd* mutants (*f*,*i,l*) is fully rescued by *prd*-PrdΔPBC (*g*,*j,m*).

*prd*, initially identified as a pair-rule gene involved in embryonic segmentation, has been shown to perform multiple functions in development, including: (i) activation of segment-polarity genes and proper segmentation of the larval cuticle [5–7]; (ii) postembryonic viability [10]; (iii) AG development [10]; and (iv) male fertility [10,12]. A *prd* transgene consisting of the entire *prd* coding region as well as the full *cis*-regulatory elements, *prd*-Prd, was able to rescue all *prd* functions in *prd* mutants (figure 1*a*) [28]. To examine the specific contribution of RED domain to Prd functions *in vivo*, we introduced another transgene, *prd*-PrdΔPBC, into *prd* mutants and examined its ability to rescue the mutant phenotypes (figure 1*a*). *prd*-PrdΔPBC replaces the Prd coding region in *prd*-Prd by a truncated version with a deletion of the RED domain (amino acids 75–125, figure 1*a*). According to the crystal structure [24] and the *in vitro* experiments [27], this deletion of the RED domain should not affect the DNA-binding property of the PAI domain [24].

The *prd* mutants used in this study are heterozygous for the deficiency *Df(2L)Prl* and the *prd^2.45^* allele, which has a 1.1 kb insertion after amino acid 45 of the PD and acts as a null allele [29]. Compared with the heterozygous control (figure 1*b*), *prd* mutants lose half of the segmental equivalents and exhibit the classical pair-rule phenotype in larval cuticle (figure 1*c*) [1]. We found that *prd*-PrdΔPBC could fully rescue the cuticle phenotype of *prd* mutants (figure 1*d*), indicating that the RED domain is not essential for the cuticle function of Prd.

Prd is required for the activation of segment polarity genes in every other parasegment in early embryos. In *prd* mutant embryos, the expression patterns of Gsb, Wg and En are abolished with a double-segment periodicity (figure 1*f*,*i*,*l*) when compared with the control (figure 1*e*,*h,k*). Consistent with its ability to rescue the *prd* mutant cuticle phenotype, *prd*-PrdΔPBC is able to fully rescue the expression patterns of Gsb, Wg and En (figure 1*g*,*j,m*), demonstrating that the RED domain is dispensable for Prd to activate the transcription of segmental polarity genes in embryonic development.

**Figure 2.**
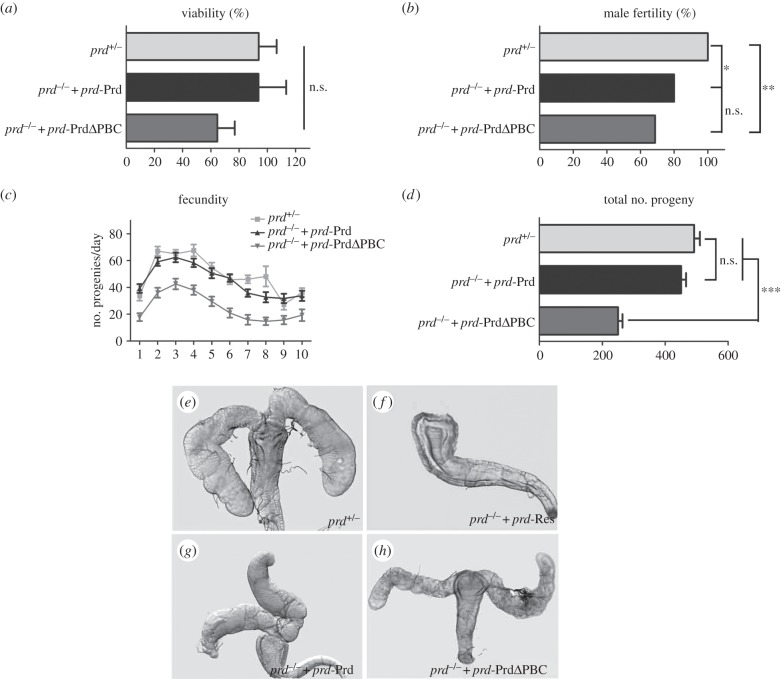
The RED domain is dispensable for the viability and male fertility functions of Prd. (*a*) *prd*-PrdΔPBC is able to rescue *prd* mutants to adulthood at a similar rate as *prd-*Prd or the endogenous *prd*. Viability is scored by the actual number over the expected number of (i) *prd^+/−^*, (ii) *prd^−/−^*+*prd*-PrdΔPBC or (iii) *prd^−/−^* + *prd*-Prd from corresponding crosses. There is no significant difference in the viability among *prd^+/−^* (92/102 expected), *prd^−/−^; prd-*Prd/+ (72/64 expected) and *prd^−/−^; prd*-PrdΔPBC/+ (31/71 expected) flies. (*b*) *prd*-PrdΔPBC rescues the fertility of *prd* mutant males to a similar extent as *prd*-Prd, but slightly lower than that of endogenous *prd*. Number of flies examined (fertile/sterile): *prd^+/−^* (*n* = 24; 24/0), *prd^−/−^; prd-*Prd/+ (*n* = 44; 35/9) and *prd^−/−^; prd*-PrdΔPBC/+ (*n* = 22; 15/7). (*c,d*) *prd* mutant males rescued by *prd*-PrdΔPBC have a reduced fecundity when compared with those rescued by *prd-*Prd or the heterozygous controls. The number of progenies produced per vial per day (*c*) and the total number of progenies for 10 days (*d*) of *prd^+/−^* (*n* = 27), *prd^−/−^; prd-*Prd/+ (*n* = 24) and *prd^−/−^; prd*-PrdΔPBC/+ (*n* = 17) are shown as mean ± s.e.m. For statistical analysis: *p < 0.05; **p < 0.01; ***p < 0.001; n.s., not significant. (*e*–*h*) Light micrographs showing AG from a 3-day-old *prd^+/−^* (*e*), *prd^−/−^; prd-*Res/+ (*f*), *prd^−/−^; prd-*Prd/+ (*g*) or *prd^−/−^; prd*-PrdΔPBC/+ (*h*) male. The loss of AG phenotype in *prd* mutant males (*f*) was rescued by *prd-*Prd (*g*) or *prd*-PrdΔPBC (*h*).

We have shown in previous work that an evolutionary allele of *prd*, *prd*-Pax3, in which the Prd mouse homologue Pax3 is placed under the control of the entire *prd cis*-regulatory region, is able to rescue larval cuticles and target segment-polarity genes expression, but not the embryonic lethality, in *prd^−^* embryos [10]. Therefore, *prd* has a viability function independent of its cuticle functions to ensure the survival of embryos to adults [9,10]. Furthermore, *prd^−^* embryos rescued by two copies of *prd*-Gsb, another evolutionary allele of *prd*, are able to develop into adulthood, yet some of the adult flies display a distorted segment phenotype in the abdominal cuticle [10], suggesting a role of Prd in regulating adult segmentation. We found that *prd*-PrdΔPBC is able to rescue the lethality in *prd* mutant embryos to a similar extent as that of *prd*-Prd, and there is no significant difference when compared with that of heterozygous controls (figure 2*a*), demonstrating that the RED domain is unnecessary for the viability function of Prd. However, *prd* mutant flies rescued by *prd*-PrdΔPBC produce a distorted segmentation phenotype in adult cuticles (electronic supplementary material, figure S1*b*,*e*), which phenocopies that of *prd* mutants rescued by *prd*-Gsb [10], suggesting that the RED domain contributes to the adult cuticle function of Prd.

### 3.2. The RED domain is dispensable for the male fertility function of Prd

Previous work reported that another transgene, *prd*-Res, in which the downstream sequence of *prd*-Prd is deleted, is sufficient to rescue *prd* mutants to adulthood, yet all males are sterile [8]. Additional studies confirmed that *prd* is required for the development of AGs, which secrete seminal fluid necessary for *Drosophila* male fertility [8,9,11,12]. The male fertility function of *prd* depends on a 0.8 kb Prd male fertility enhancer (PMFE) located in the *prd* downstream region [12], as *prd* mutant males rescued by *prd*-Res, which does not include PMFE, produces a loss of AG phenotype (figure 2*f*) [9,12]. We then examined the rescue ability of *prd*-PrdΔPBC in AG development and male fertility in *prd* mutants. We found that *prd*-PrdΔPBC could rescue AG development to a similar extent as that of *prd*-Prd (figure 2*g,h*). Furthermore, about 70% of *prd* mutant males rescued by *prd*-PrdΔPBC are fertile, which is not statistically different from the case with *prd*-Prd (figure 2*b*). Hence, we conclude that the RED domain is dispensable for the male fertility function of Prd.

However, *prd^−^* males rescued by *prd*-PrdΔPBC display a significantly reduced fecundity as they produce far less progen than the heterozygous controls or *prd^−^* males rescued by *prd*-Prd (figure 2*c,d*), suggesting the RED domain, though not necessary, still contributes to the male fertility. Prd is known to play a later function in AG maturation by regulating the expression of a variety of AG products, including SP and Acp26Aa, which stimulate female egg laying [12]. As shown below, these factors are significantly reduced in the AGs of *prd* mutant males rescued by *prd*-PrdΔPBC, which might account for the reduced number of eggs/progeny produced by their mates.

In addition, we also noted that *prd^−^* males rescued by *prd*-PrdΔPBC exhibited a lower mating success rate compared with those rescued by *prd*-Prd (electronic supplementary material, figure S2*a*), yet there is no significant difference in the climbing ability between these two groups of flies (electronic supplementary material, figure S2*b*), suggesting factors other than the locomotion skill are responsible for the different copulation outcome.

### 3.3. The RED domain is necessary for males to elicit post-mating response in mated females

Reproduction is one of the fundamental missions of life. Hence, many species have evolved an intricate variety of mechanisms to guarantee the success of procreation. In most insects, including *Drosophila melanogaster,* male-derived substances transferred during mating induce significant behavioural changes, so-called PMR, leading to an increased egg laying and decreased sexual receptivity in their mated partners [15,30–32].

**Figure 3.**
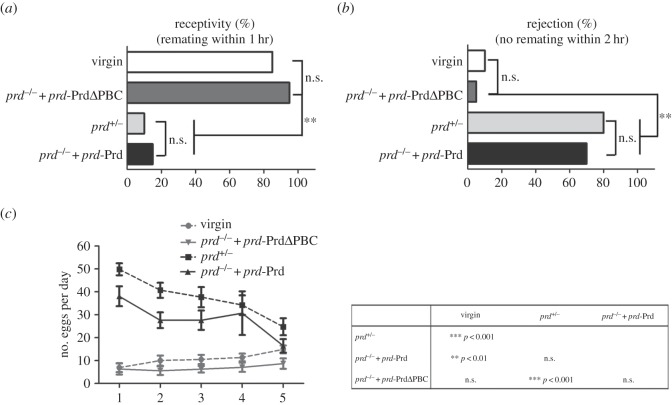
The RED domain is imperative for female post-mating response (PMR). (*a*,*b*) Females mated with *prd^−/−^; prd*-PrdΔPBC/+ males exhibit a virgin-like behaviour with a high receptivity (*a*) and low rejection (*b*) to second mating, whereas those that copulated with the heterozygous controls or *prd^−/−^; prd-*Prd/+ males exhibit an opposite behaviour with a low receptivity (*a*) and high rejection (*b*). (*a*) The percentage of virgin females (17/20) mated to naive *w^1118^* males within 1 h or the percentage of non-virgin females previously mated to *prd^+/−^*(2/20), *prd^−/−^; prd*-PrdΔPBC/+ (19/20) or *prd^−/−^; prd-*Prd/+ (3/20) males successfully re-mating within 1 h. (*b*) The percentage of virgin females (2/20) not mated within 2 h or the percentage of non-virgin females previously mated to *prd^+/−^*(16/20), *prd^−/−^; prd*-PrdΔPBC/+ (1/20) or *prd^−/−^; prd-*Prd/+ (14/20) males not re-mated within 2 h. For statistical analysis: *p < 0.05; **p < 0.01; n.s., not significant. (*c*) Egg-laying of virgin females or females mated to (i) *prd^+/−^*, (ii) *prd^−/−^; prd-*Prd/+ or (iii) *prd^−/−^; prd*-PrdΔPBC/+ males (*n* = 20, respectively). Females mated with heterozygous controls or *prd^−/−^; prd-*Prd/+ show dramatic increase in egg-laying that lasts for a few days with a gradual reduction. However, females mated with *prd^−/−^; prd*-PrdΔPBC/+ males fail to trigger the increased oviposition and lay few eggs per day, as do virgin females. The statistical analysis of egg laying using one-way ANOVA followed by Bonferroni's multiple comparison test is shown on the right. Significant differences are indicated as *p < 0.05; **p < 0.01; ***p < 0.001.

**Figure 4.**
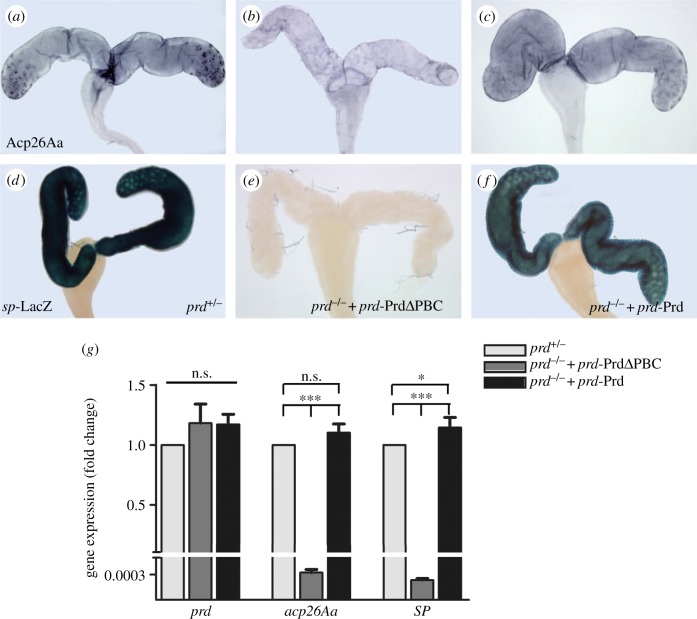
The RED domain is required for the expression of Acp26Aa and SP in the AG. The expression of Acp26Aa (*a*–*c*) and SP (*d*–*f*) in AG from a 3-day-old *prd^+/−^* (*a* or *d*), *prd^−/−^; prd*-PrdΔPBC/+ (*b* or *e*) or *prd^−/−^; prd*-Prd/+ (*c* or *f*) male. The expression of Acp26Aa (*b*) or SP (*e*) in *prd* mutant males rescued by *prd*-PrdΔPBC was undetectable when compared with controls (*a,d*) or *prd* mutants rescued by *prd*-Prd (*c,f*). (*g*) qRT-PCR assay showing the transcription level of *prd*, *acp26A* and *SP* in AG of indicated genotypes (*n* = 10 per genotype). Significant differences are indicated as *p < 0.05; *** *p* < 0.001, n.s., not significant.

Virgin females exhibit a high receptivity (figure 3*a*) and low rejection (figure 3*b*) towards males, and lay less than 10 eggs per day (figure 3*c*). The females show dramatically increased oviposition (up to about 50 eggs within 24 h) after mating to control males, and maintain this rate in the next few days with a gradual reduction (figure 3*c*). Meanwhile, mated females display a low receptivity (figure 3*a*) and high rejection (figure 3*b*) towards males after first copulation. However, females mated to *prd* mutant males rescued by *prd*-PrdΔPBC behave like virgins with low egg-laying (figure 3*c*), high receptivity (figure 3*a*) and low rejection (figure 3*b*). In contrast, *prd* mutant males rescued by *prd*-Prd are able to elicit PMR in their mates to a similar extent as control males (figure 3*a*–*c*). To rule out the possibility that the rescue failure of *prd*-PrdΔPBC is due to its lower expression level, we checked the transgene expression in male AGs by qRT-PCR, and found that *prd*-PrdΔPBC was expressed at a level comparable with that of *prd*-Prd or endogenous *prd* (figure 4*g*). Furthermore, adding another copy of *prd*-PrdΔPBC to *prd* mutant males does not improve PMR in their mates (electronic supplementary material, figure S3). Together, these data suggest that the RED domain is necessary for Prd to induce PMR in mated females.

### 3.4. The RED domain is necessary for Prd to activate the expression of Acp26Aa and sex peptide in accessory glands

Female PMRs have been correlated with substances derived from the male AG [32,33], and among them Acp26Aa and SP have been extensively studied. Meanwhile, a dual role of *prd* in male AG development has been characterized [12]. Prd is required at an early developmental stage to promote cell proliferation and later for the expression of secretions, including Acp26Aa and SP, to ensure the maturation of the AG [12]. Acp26Aa stimulates the release of oocytes from the ovary [16], which is the first step of the egg-laying process essential for the initial increase of oviposition in females after mating [34]. Recent research indicated that Acp26Aa increases ovulation and egg laying through the OA neuronal signalling [35]. SP, a key component of AG secretions that is transferred into females with sperm, downregulates the excitability of SP sensory neurons (SPSNs) in the female reproductive tract and their input onto SAG neurons of the abdominal ganglion, which results in an increased oviposition and reduced sexual receptivity [36–38]. SP also binds to the sperm and induces a prolonged PMR in females by a gradual release from the sperm stored in mated females [39].

Owing to the virgin-like behaviour of females copulated to *prd* mutant males rescued by *prd*-PrdΔPBC (figure 3*a*–*c* and electronic supplementary material, figure S3), we examined the expression of Acp26Aa and SP in male AGs. In control AGs, Acp26Aa is expressed in the whole organ (figure 4*a*), whereas SP is detected only in the main cells by using a *sp*-LacZ reporter transgene (figure 4*d*) [12]. Although *prd*-PrdΔPBC is able to rescue AG development and male fertility in *prd* mutant males, it fails to rescue the expression pattern of Acp26Aa (figure 4*b*) and SP (figure 4*e*), when compared with *prd*-Prd (figure 4*c,f*). These results were confirmed by qRT-PCR assay (figure 4*g*). Thus, we conclude that the RED domain is indispensable for Prd to activate the expression of AG secretions, which are essential for PMR but not for male fertility.

Gsb, first identified as a member of segment-polarity genes [1] and activated by Prd during embryogenesis [3,40], is required for the establishment of positional information along the anterior–posterior axis in the epidermis. In addition, Gsb also plays an important role in the *Drosophila* central nervous system (CNS), including the specification of embryonic neuronal cell fate [41–45] and the maintenance of postembryonic synaptic homeostasis [46]. We previously found that Gsb is specifically expressed in the secondary cells located in the distal region of each AG tube (electronic supplementary material, figure S4*a*) [12], though the function of Gsb in the AG has remained unknown. We found that Gsb expression was rescued in *prd^−^* males by *prd*-Prd, but not *prd*-PrdΔPB (electronic supplementary material, figure S4*b,c*), which is confirmed by qRT-PCR assay (electronic supplementary material, figure S4*d*). Thus, the RED domain is also required for Prd to activate gsb expression in the AG.

It was proposed that the RED domain within the C-terminal of PD, though containing a functional DNA-binding motif, is dispensable for the physiological functions of Prd. In this study, we demonstrate that the RED domain is dispensable for most functions of Prd in development, yet it is specifically required for Prd to activate the expression of AG products Acp26Aa and SP, and thus to trigger PMR in mated females. However, the underlying mechanism by which the RED domain modulates Acp26Aa and SP expression, either through a direct transcriptional activation by binding to the *cis*-regulatory region or indirectly, requires further investigation.

## 4. Material and methods

### 4.1. Fly strains

All flies were raised on standard *Drosophila* media and maintained at 25°C. *prd^2.45^*, *Df(2L)Prl, prd*-Prd [9] and *prd*-Res [12] were described previously. *Prd*-PrdΔPBC was produced by inserting the coding sequence from hs-PrdΔPBC [47] into *prd*-0 [10]. The plasmid was injected with Δ2–3 helper plasmid into *ry^506^* embryos and *ry^+^* transformants were selected. All the strains are in the same *ry^506^* background.

### 4.2. Immunostaining of embryos and cuticle preparation

Embryo collection, fixation and immunostaining were carried out as described [48]. Double-labelling of embryos for β-galactosidase and Gsb, Wg or En protein was performed as described [49]. Cuticles were prepared essentially as described [8].

### 4.3. Dissection, immunostaining and X-Gal staining of male accessory glands

AGs were dissected [11] and stained with anti-Acp26Aa [50] and anti-Gsb [45] as described. X-Gal staining was performed according to Bertram *et al.* [13].

### 4.4. Male fertility, fecundity, egg laying and receptivity assay

All flies were aged for 3 days after eclosion and then analysed. For male fertility assay, *w^1118^* females, which were successfully mated by (i) *prd^2.45^/+*, (ii) *Df(2L)Prl*/*prd^2.45^; prd*-PrdΔPBC/+ or (iii) *Df(2L)Prl*/*prd^2.45^*; *prd*-Prd/+ males, were maintained separately on standard medium. The male was scored as fertile or sterile depending on the presence or absence of offspring, respectively. The data were calculated and presented as the ratio of fertile males. For male fecundity assay, each cross was of two virgin *w^1118^* females placed by one (i) *prd^2.45^/+*, (ii) *Df(2L)Prl*/*prd^2.45^; prd*-PrdΔPBC/+ or (iii) *Df(2L)Prl*/*prd^2.45^; prd*-Prd/+ male and transferred into fresh vials with standard media every 24 h for 10 days. The number of progenies produced per vial was scored (figure 2*c*), and also the total number in 10 days was calculated (figure 2*d*). For egg laying, virgin or none virgin (successfully mated by the indicated males) *w^1118^* females were transferred into fresh vials with standard media every 24 h and allowed to lay eggs for 5 days. The number of eggs laid by one individual female was scored every day. For receptivity assay, virgin or none virgin (successfully mated by the test males) *w^1118^* females were housed individually for 24 h after first mating and then examined in a receptivity assay with naive *w^1118^* males. The receptivity was classified as remating within 1 h, and rejection was categorized as no remating within 2 h, respectively. Final data were calculated as the ratio of each classification.

### 4.5. Rapid iterative negative geotaxis (climbing) assay

A modified version of Nichols's climbing assay was used [51] (electronic supplementary material, figure 2*b*). Briefly, 2-day-old flies were collected separately by gender within 24 h after eclosion. Ten to 15 flies per genotype were placed in a vertical vial (20 cm height, 2.5 cm diameter), and the vials were tapped at the bottom regularly. A picture was taken 5 s after each tapping and the average heights of flies climbing were calculated. Each analysis was repeated five times with 60 s resting intervals. The number of flies tested per genotype was *n* = 30 for females or *n* = 15 for males.

### 4.6. Statistical analysis

Statistical analysis of viability, male fecundity and egg laying assays was performed using one-way ANOVA followed by Bonferroni's multiple comparison test. Statistical analysis of male fertility, receptivity and rejection assays was performed using Fisher's exact test.

### 4.7. qRT-PCR

About 10 AGs were collected from 1-day-old males of indicated genotypes, and RT-PCR was performed as previously described [52]. Total RNA was extracted using the Ambion PureLink RNA mini kit according to the manufacturer's instructions. Primers used for qRT-PCR are as follows:

**Table:** 

*actin88F*	sense 5′- ATCGAGCACGGCATCATCAC-3′
	antisense 5′- CACGCGCAGCTCGTTGTA-3′
*paired*	sense 5′-CAGTCACGCCAACATTTCCG -3′
	antisense 5′-ACCCGGCATTATGTAGCTGG-3′
*Acp26Aa*	sense 5′-TCAAGGATCCAATCAAAGTGC-3′
	antisense 5′-ACGTTGGCTTCCTGAAACTG-3′
*SP*	sense 5′-GAATGGCCGTGGAATAGGAA-3′
	antisense 5′-GGCACCACTTATCACGAGGATT-3′
*gsb*	sense 5′-ATGACACCCTACTTTGGCGG-3′
	antisense 5′-TGCTGCCATCTCCACGATTT-3′

## Supplementary Material

Supplementary figures

## Supplementary Material

Supplementary figure legends

## Data accessibility

The datasets supporting this article have been uploaded as part of the electronic supplementary material.

## References

[1] Nusslein-Volhard C Wieschaus E 1980 Mutations affecting segment number and polarity in *Drosophila*. Nature 287, 795–801 (doi:10.1038/287795a0) 6776413 10.1038/287795a0

[2] Baumgartner S Noll M 1990 Network of interactions among pair-rule genes regulating paired expression during primordial segmentation of *Drosophila*. Mech. Dev. 33, 1–18 (doi:10.1016/0925-4773(90)90130-E) 1982920 10.1016/0925-4773(90)90130-e

[3] Ingham PW Martinez Arias A 1992 Boundaries and fields in early embryos. Cell 68, 221–235 (doi:10.1016/0092-8674(92)90467-Q) 1346367 10.1016/0092-8674(92)90467-q

[4] St Johnston D Nusslein-Volhard C 1992 The origin of pattern and polarity in the *Drosophila* embryo. Cell 68, 201–219 (doi:10.1016/0092-8674(92)90466-P) 1733499 10.1016/0092-8674(92)90466-p

[5] DiNardo S O'Farrell PH 1987 Establishment and refinement of segmental pattern in the *Drosophila* embryo: spatial control of engrailed expression by pair-rule genes. Genes Dev. 1, 1212–1225 (doi:10.1101/gad.1.10.1212) 3123316 10.1101/gad.1.10.1212

[6] Ingham PW Baker NE Martinez-Arias A 1988 Regulation of segment polarity genes in the *Drosophila* blastoderm by *fushi tarazu* and *even skipped*. Nature 331, 73–75 (doi:10.1038/331073a0) 2893285 10.1038/331073a0

[7] Bopp D Jamet E Baumgartner S Burri M Noll M 1989 Isolation of two tissue-specific *Drosophila* paired box genes, Pox meso and Pox neuro. EMBO J. 8, 3447–3457. 2573516 10.1002/j.1460-2075.1989.tb08509.x PMC401500

[8] Bertuccioli C Fasano L Jun S Wang S Sheng G Desplan C 1996 *In vivo* requirement for the paired domain and homeodomain of the paired segmentation gene product. Development 122, 2673–2685. 8787742 10.1242/dev.122.9.2673

[9] Xue L Li X Noll M 2001 Multiple protein functions of Paired in *Drosophila* development and their conservation in the Gooseberry and Pax3 homologs. Development 128, 395–405. 11152638 10.1242/dev.128.3.395

[10] Xue L Noll M 1996 The functional conservation of proteins in evolutionary alleles and the dominant role of enhancers in evolution. EMBO J. 15, 3722–3731. 8670876 PMC452034

[11] Xue L Noll M 2000 *Drosophila* female sexual behavior induced by sterile males showing copulation complementation. Proc. Natl Acad. Sci. USA 97, 3272–3275 (doi:10.1073/pnas.97.7.3272) 10725377 10.1073/pnas.060018897 PMC16228

[12] Xue L Noll M 2002 Dual role of the *Pax* gene paired in accessory gland development of *Drosophila*. Development 129, 339–346. 11807027 10.1242/dev.129.2.339

[13] Bertram MJ Akerkar GA Ard RL Gonzalez C Wolfner MF 1992 Cell type-specific gene expression in the *Drosophila melanogaster* male accessory gland. Mech. Dev. 38, 33–40 (doi:10.1016/0925-4773(92)90036-J) 1525037 10.1016/0925-4773(92)90036-j

[14] Chen PS 1996 The accessory gland proteins in male *Drosophila*: structural, reproductive, and evolutionary aspects. Experientia 52, 503–510 (doi:10.1007/BF01969718) 8698082 10.1007/BF01969718

[15] Wolfner MF 1997 Tokens of love: functions and regulation of *Drosophila* male accessory gland products. Insect Biochem. Mol. Biol. 27, 179–192 (doi:10.1016/S0965-1748(96)00084-7) 9090115 10.1016/s0965-1748(96)00084-7

[16] Heifetz Y Lung O Frongillo EA Jr Wolfner MF 2000 The *Drosophila* seminal fluid protein Acp26Aa stimulates release of oocytes by the ovary. Curr. Biol. 10, 99–102 (doi:10.1016/S0960-9822(00)00288-8) 10662669 10.1016/s0960-9822(00)00288-8

[17] Ravi Ram K Ramesh SR 2003 Male accessory gland proteins in *Drosophila*: a multifaceted field [corrected]. Indian J. Exp. Biol. 41, 1372–1383. 15320489

[18] Noll M 1993 Evolution and role of *Pax* genes. Curr. Opin. Genet. Dev. 3, 595–605 (doi:10.1016/0959-437X(93)90095-7) 8241771 10.1016/0959-437x(93)90095-7

[19] Wang QY Fang WH Krupinski J Kumar S Slevin M Kumar P 2008 *Pax* genes in embryogenesis and oncogenesis. J. Cell. Mol. Med. 12, 2281–2294 (doi:10.1111/j.1582-4934.2008.00427.x) 18627422 10.1111/j.1582-4934.2008.00427.x PMC4514106

[20] Thompson JA Ziman M 2011 *Pax* genes during neural development and their potential role in neuroregeneration. Prog. Neurobiol. 95, 334–351 (doi:10.1016/j.pneurobio.2011.08.012) 21930183 10.1016/j.pneurobio.2011.08.012

[21] Underhill DA 2012 PAX proteins and fables of their reconstruction. Crit. Rev. Eukar. Gene 22, 161–177 (doi:10.1615/CritRevEukarGeneExpr.v22.i2.70) 10.1615/critreveukargeneexpr.v22.i2.70 22856433

[22] Treisman J Harris E Desplan C 1991 The paired box encodes a second DNA-binding domain in the paired homeo domain protein. Genes Dev. 5, 594–604 (doi:10.1101/gad.5.4.594) 1672661 10.1101/gad.5.4.594

[23] Bopp D Burri M Baumgartner S Frigerio G Noll M 1986 Conservation of a large protein domain in the segmentation gene *paired* and in functionally related genes of *Drosophila*. Cell 47, 1033–1040 (doi:10.1016/0092-8674(86)90818-4) 2877747 10.1016/0092-8674(86)90818-4

[24] Xu W Rould MA Jun S Desplan C Pabo CO 1995 Crystal structure of a paired domain-DNA complex at 2.5 A resolution reveals structural basis for Pax developmental mutations. Cell 80, 639–650 (doi:10.1016/0092-8674(95)90518-9) 7867071 10.1016/0092-8674(95)90518-9

[25] Xu HE Rould MA Xu W Epstein JA Maas RL Pabo CO 1999 Crystal structure of the human Pax6 paired domain-DNA complex reveals specific roles for the linker region and carboxy-terminal subdomain in DNA binding. Genes Dev. 13, 1263–1275 (doi:10.1101/gad.13.10.1263) 10346815 10.1101/gad.13.10.1263 PMC316729

[26] Sun H Rodin A Zhou Y Dickinson DP Harper DE Hewett-Emmett D Li W.H 1997 Evolution of paired domains: isolation and sequencing of jellyfish and hydra *Pax* genes related to *Pax-5* and *Pax-6*. Proc. Natl Acad. Sci. USA 94, 5156–5161 (doi:10.1073/pnas.94.10.5156) 9144207 10.1073/pnas.94.10.5156 PMC24648

[27] Jun S Desplan C 1996 Cooperative interactions between paired domain and homeodomain. Development 122, 2639–2650. 8787739 10.1242/dev.122.9.2639

[28] Gutjahr T Vanario-Alonso CE Pick L Noll M 1994 Multiple regulatory elements direct the complex expression pattern of the *Drosophila* segmentation gene *paired*. Mech. Dev. 48, 119–128 (doi:10.1016/0925-4773(94)90021-3) 7873402 10.1016/0925-4773(94)90021-3

[29] Frigerio G Burri M Bopp D Baumgartner S Noll M 1986 Structure of the segmentation gene *paired* and the *Drosophila* PRD gene set as part of a gene network. Cell 47, 735–746 (doi:10.1016/0092-8674(86)90516-7) 2877746 10.1016/0092-8674(86)90516-7

[30] Kubli E 2003 Sex-peptides: seminal peptides of the *Drosophila* male. Cell. Mol. Life Sci. 60, 1689–1704 (doi:10.1007/s00018-003-3052) 14504657 10.1007/s00018-003-3052 PMC11146071

[31] Yang CH Rumpf S Xiang Y Gordon MD Song W Jan LY Jan YN 2009 Control of the postmating behavioral switch in *Drosophila* females by internal sensory neurons. Neuron 61, 519–526 (doi:10.1016/j.neuron.2008.12.021) 19249273 10.1016/j.neuron.2008.12.021 PMC2748846

[32] Haussmann IU Hemani Y Wijesekera T Dauwalder B Soller M 2013 Multiple pathways mediate the sex-peptide-regulated switch in female *Drosophila* reproductive behaviours. Proc. R. Soc. B 280, 20131938 (doi:10.1098/rspb.2013.1938) 10.1098/rspb.2013.1938 PMC3790487 24089336

[33] Smith DT Hosken DJ Ffrench-Constant RH Wedell N 2009 Variation in sex peptide expression in *D. melanogaster*. Genet. Res. 91, 237–242 (doi:10.1017/S0016672309000226) 10.1017/S0016672309000226 19640319

[34] Herndon LA Wolfner MF 1995 A *Drosophila* seminal fluid protein, Acp26Aa, stimulates egg laying in females for 1 day after mating. Proc. Natl Acad. Sci. USA 92, 10 114–10 118 (doi:10.1073/pnas.92.22.10114) 10.1073/pnas.92.22.10114 PMC40746 7479736

[35] Rubinstein CD Wolfner MF 2013 *Drosophila* seminal protein ovulin mediates ovulation through female octopamine neuronal signaling. Proc. Natl Acad. Sci. USA 110, 17 420–17 425 (doi:10.1073/pnas.1220018110) 10.1073/pnas.1220018110 PMC3808635 24101486

[36] Hasemeyer M Yapici N Heberlein U Dickson BJ 2009 Sensory neurons in the *Drosophila* genital tract regulate female reproductive behavior. Neuron 61, 511–518 (doi:10.1016/j.neuron.2009.01.009) 19249272 10.1016/j.neuron.2009.01.009

[37] Rezaval C Pavlou HJ Dornan AJ Chan YB Kravitz EA Goodwin SF 2012 Neural circuitry underlying *Drosophila* female postmating behavioral responses. Curr. Biol. 22, 1155–1165 (doi:10.1016/j.cub.2012.04.062) 22658598 10.1016/j.cub.2012.04.062 PMC3396843

[38] Feng K Palfreyman MT Hasemeyer M Talsma A Dickson BJ 2014 Ascending SAG neurons control sexual receptivity of *Drosophila* females. Neuron 83, 135–148 (doi:10.1016/j.neuron.2014.05.017) 24991958 10.1016/j.neuron.2014.05.017

[39] Peng J Chen S Busser S Liu H Honegger T Kubli E 2005 Gradual release of sperm bound sex-peptide controls female postmating behavior in *Drosophila*. Curr. Biol. 15, 207–213 (doi:10.1016/j.cub.2005.01.034) 15694303 10.1016/j.cub.2005.01.034

[40] Bouchard M St-Amand J Cote S 2000 Combinatorial activity of pair-rule proteins on the *Drosophila gooseberry* early enhancer. Dev. Biol. 222, 135–146 (doi:10.1006/dbio.2000.9702) 10885752 10.1006/dbio.2000.9702

[41] Bhat KM van Beers EH Bhat P 2000 Sloppy paired acts as the downstream target of Wingless in the *Drosophila* CNS and interaction between sloppy paired and gooseberry inhibits sloppy paired during neurogenesis. Development 127, 655–665. 10631185 10.1242/dev.127.3.655

[42] Deshpande N Dittrich R Technau GM Urban J 2001 Successive specification of *Drosophila* neuroblasts NB 6–4 and NB 7–3 depends on interaction of the segment polarity genes *wingless, gooseberry *and* naked cuticle*. Development 128, 3253–3261. 11546742 10.1242/dev.128.17.3253

[43] Duman-Scheel M Li X Orlov I Noll M Patel NH 1997 Genetic separation of the neural and cuticular patterning functions of gooseberry. Development 124, 2855–2865. 9247329 10.1242/dev.124.15.2855

[44] Skeath JB Zhang Y Holmgren R Carroll SB Doe CQ 1995 Specification of neuroblast identity in the *Drosophila* embryonic central nervous system by *gooseberry-distal*. Nature 376, 427–430 (doi:10.1038/376427a0) 7630418 10.1038/376427a0

[45] Gutjahr T Patel NH Li X Goodman CS Noll M 1993 Analysis of the gooseberry locus in *Drosophila* embryos: gooseberry determines the cuticular pattern and activates gooseberry neuro. Development 118, 21–31. 8375335 10.1242/dev.118.1.21

[46] Marie B Pym E Bergquist S Davis GW 2010 Synaptic homeostasis is consolidated by the cell fate gene *gooseberry*, a *Drosophila* *pax3/7* homolog. J. Neurosci. 30, 8071–8082 (doi:10.1523/JNEUROSCI.5467-09.2010) 20554858 10.1523/JNEUROSCI.5467-09.2010 PMC3291498

[47] Cai J Lan Y Appel LF Weir M 1994 Dissection of the *Drosophila* paired protein: functional requirements for conserved motifs. Mech. Dev. 47, 139–150 (doi:10.1016/0925-4773(94)90086-8) 7811637 10.1016/0925-4773(94)90086-8

[48] Gutjahr T Frei E Noll M 1993 Complex regulation of early paired expression: initial activation by gap genes and pattern modulation by pair-rule genes. Development 117, 609–623. 8330531 10.1242/dev.117.2.609

[49] Lawrence PA Johnston P Macdonald P Struhl G 1987 Borders of parasegments in *Drosophila* embryos are delimited by the *fushi tarazu* and *even-skipped* genes. Nature 328, 440–442 (doi:10.1038/328440a0) 2886916 10.1038/328440a0

[50] Monsma SA Harada HA Wolfner MF 1990 Synthesis of two *Drosophila* male accessory gland proteins and their fate after transfer to the female during mating. Dev. Biol. 142, 465–475 (doi:10.1016/0012-1606(90)90368-S) 2257979 10.1016/0012-1606(90)90368-s

[51] Nichols CD Becnel J Pandey UB 2012 Methods to assay *Drosophila* behavior. J. Visual. Exp. 7, pii. 3795 (doi:10.3791/3795) 22433384 10.3791/3795 PMC3671839

[52] Wang MC Bohmann D Jasper H 2003 JNK signaling confers tolerance to oxidative stress and extends lifespan in *Drosophila*. Dev. Cell 5, 811–816 (doi:10.1016/S1534-5807(03)00323-X) 14602080 10.1016/s1534-5807(03)00323-x

