# Supplementary figures and images for: The RED domain of Paired is specifically required for Drosophila accessory gland maturation
Source: Open Biol. 2015 Feb 18;5(2):140179. doi: 10.1098/rsob.140179 (PMC4345280; doi:10.1098/rsob.140179)

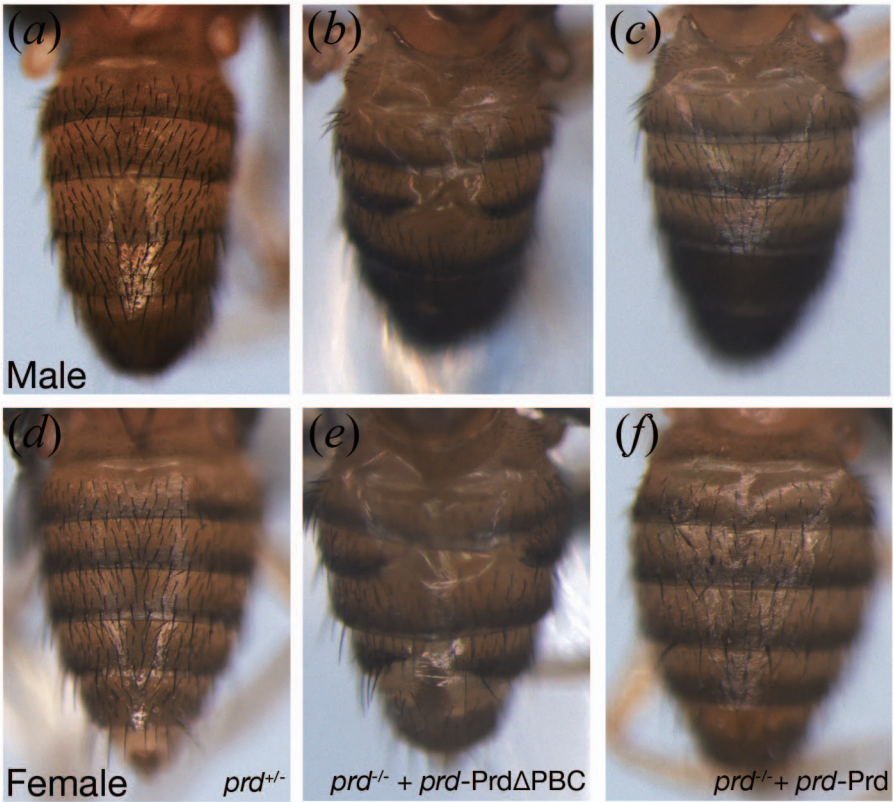

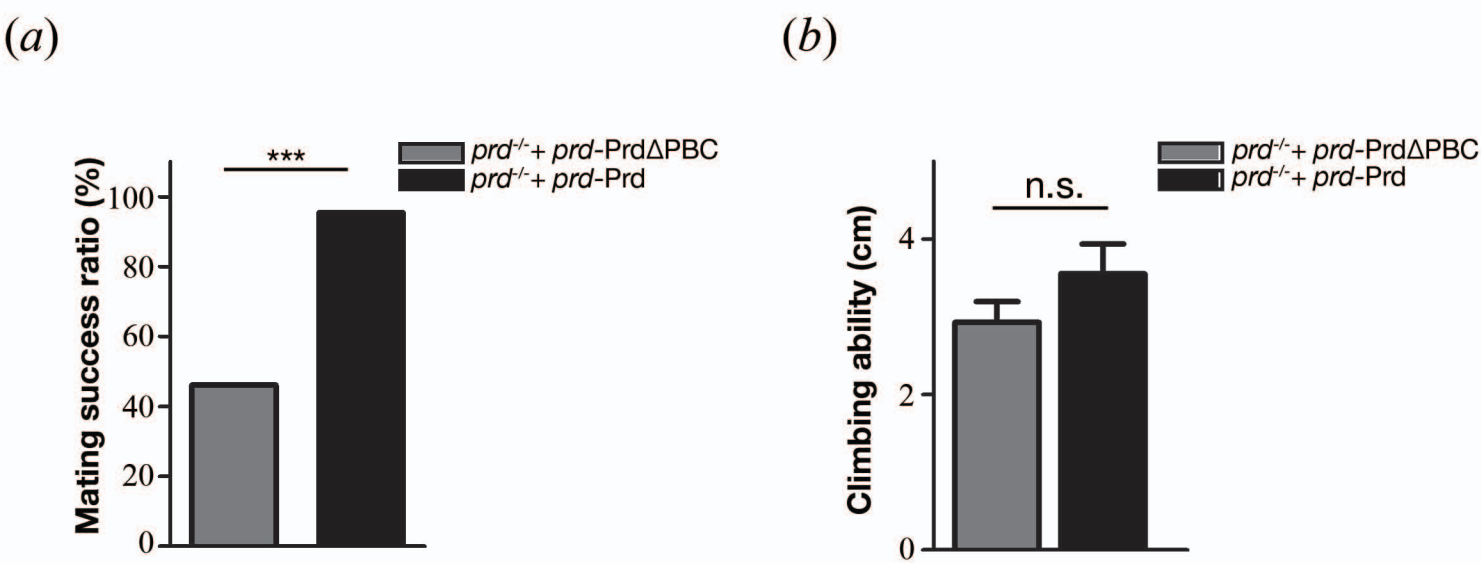

(a)

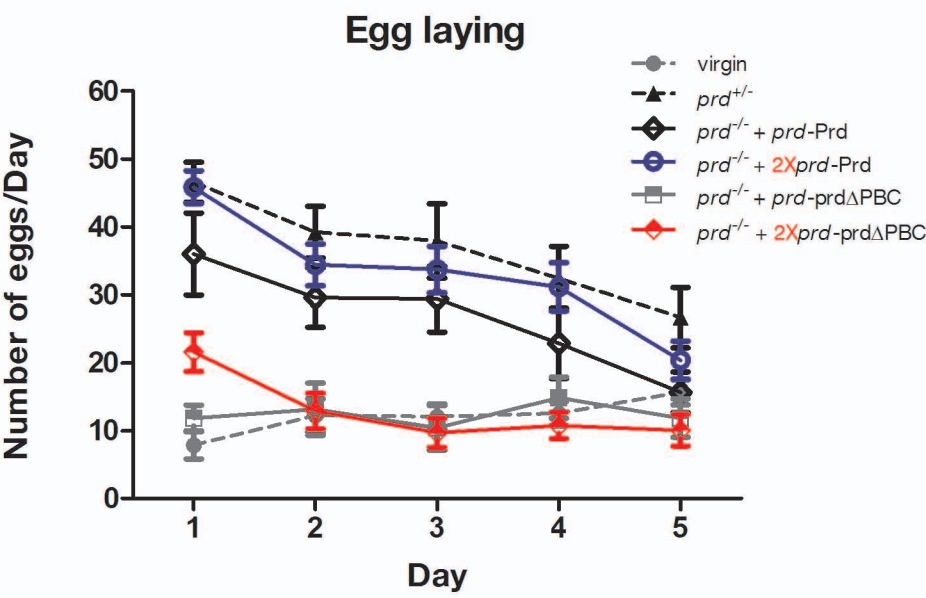

(b)

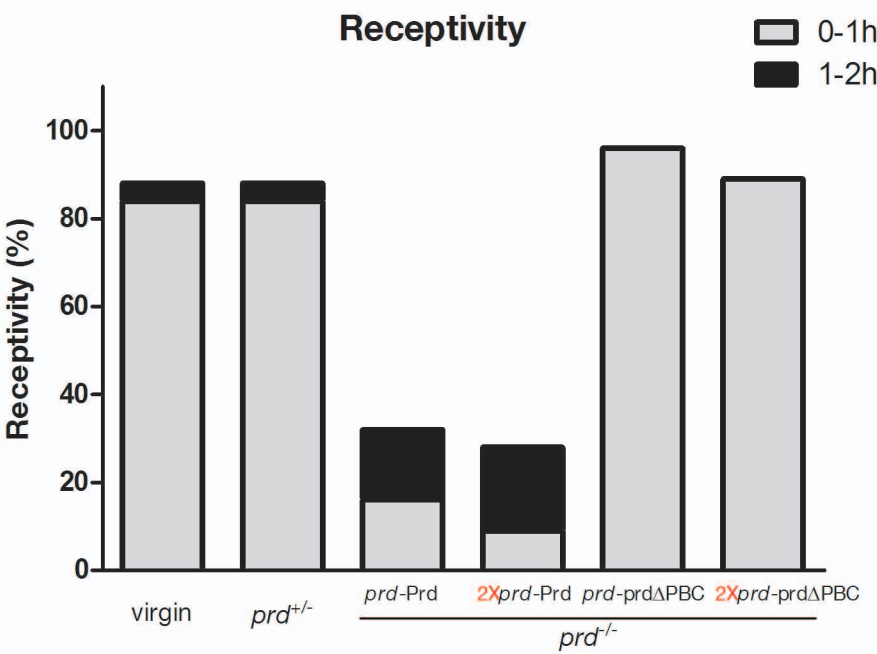

Li et al., Figure S4

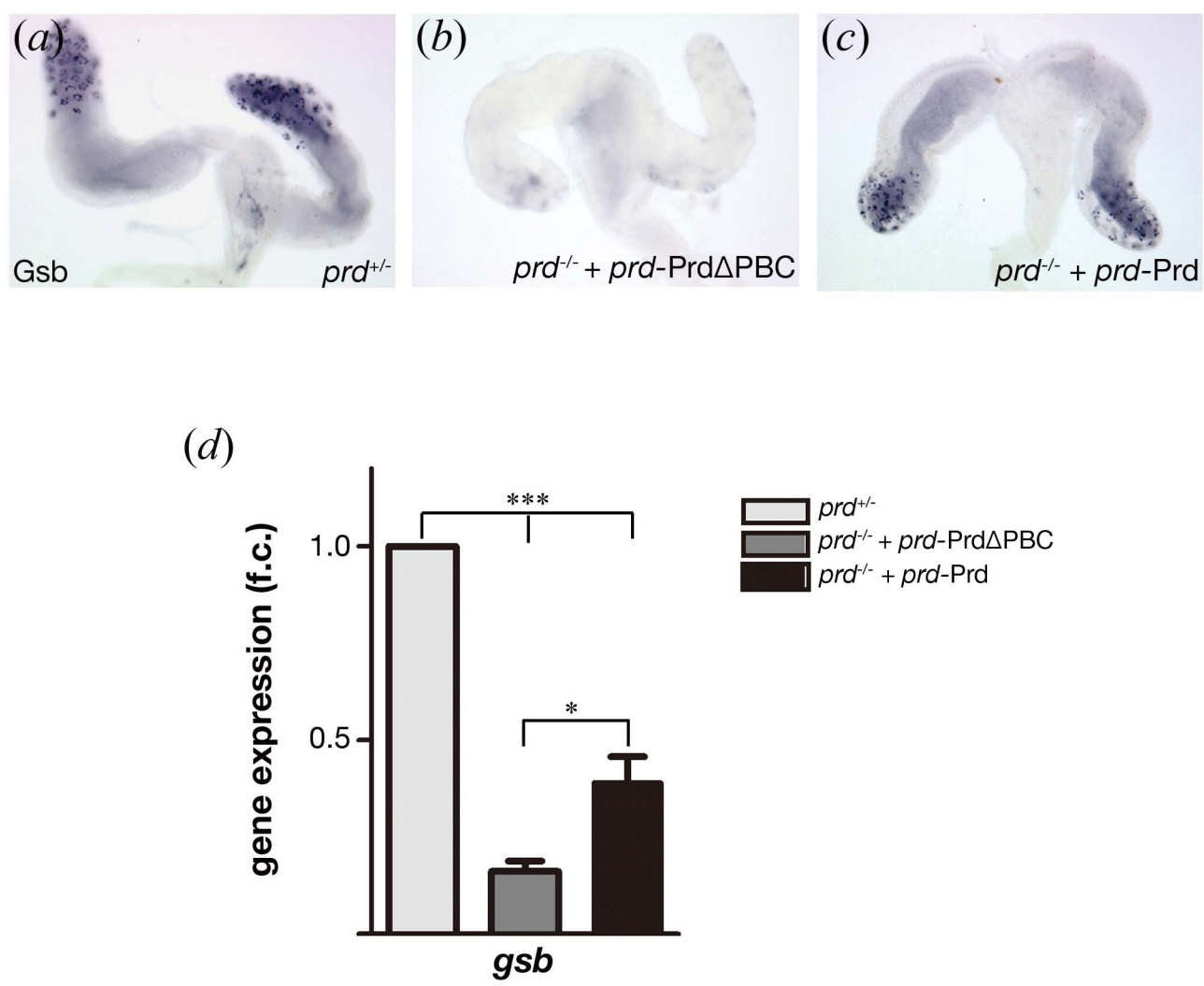

Supplement: Supplementary figures [file rsob140179supp1.pdf]
