# Supplementary material for: The RED domain of Paired is specifically required for Drosophila accessory gland maturation
Source: Open Biol. 2015 Feb 18;5(2):140179. doi: 10.1098/rsob.140179 (PMC4345280; doi:10.1098/rsob.140179)
Supplement: Supplementary figure legends [file rsob140179supp2.docx]

**Supplementary Information**

**Figure S1. *prd*-PrdΔPBC fails to rescue the adult cuticular segmentation in *prd* mutants.**

Light micrographs showing an adult male’s (***a***-***c***) or a female’s (***d***-***f***) abdominal cuticle of *prd^+/-^* (***a*** and ***d***), *prd^-/-^; prd*-PrdΔPBC/*+* (***b*** and ***e***), or *prd^-/-^; prd*-Prd /*+* (***c*** and ***f***). *prd* mutant flies rescued by *prd*-PrdΔPBC display a distorted abdominal phenotype (***b*** and ***e***) as compared with heterozygous controls (***a*** and ***d***) or *prd* mutant flies rescued by *prd*-Prd (***c*** and ***f***).

**Figure S2. The RED domain contributes to male’s mating success but not locomotion ability.**

(a) Male’s mating success ratio of *prd^-/-^; prd*-PrdΔPBC/*+* (12/26) and *prd^-/-^; prd*-Prd /*+* (41/43) were calculated by the number of males successfully mated to 3-day-old virgin females within 2 hours over the total number of tested males per genotype. All males are 3-day-old and had been kept individually after eclosion. *prd* mutant males rescued by *prd*-PrdΔPBC have a lower mating success as compared with those of *prd*-Prd. Fisher’s exact test was used for each statistical analysis. Significant differences are indicated as ***, P<0.001. (***b***) There is no significant difference in climbing ability between *prd^-/-^; prd*-PrdΔPBC/*+* males and *prd^-/-^; prd*-Prd /*+* males. One-way ANOVA followed by Bonferroni’s multiple comparison test was used for the statistical analysis. Significant differences are indicated as n.s., not significant.

**Figure S3. *prd* mutant males rescued by two copies of *prd*-PrdΔPBC fail to elicit PMR in mated females.**

Egg laying (***a***) and receptivity (***b***) of virgin or females mated to heterozygous *prd* males, *prd* mutant males rescued by one or two copies of *prd*­-Prd or *prd*-PrdΔPBC. *prd* mutant males rescued by two copies of *prd*-PrdΔPBC, just like those rescued by on copy, fail to elicit PMR in females. Thus, the absence of RED domain, but not the protein dosage, accounts for PrdΔPBC’s failure to induce PMR in females.

**Figure S4. The RED domain is required for the expression of Gsb.**

(***a***-***c***) The expression pattern of Gsb in AG from a 3-day-old *prd^+/-^* (***a***), *prd^-/-^; prd*-PrdΔPBC/*+* (***b***) or *prd^-/-^; prd*-Prd/*+* (***c***) male. The expression of Gsb in *prd* mutant males rescued by *prd*-PrdΔPBC was undetectable (***b***) as compared with controls (***a***) or *prd* mutants rescued by *prd*-Prd (***c***). (***g***) qRT-PCR assay showing the transcription level of *gsb* in AG of indicated genotypes (n=10 for each genotype). Significant differences are indicated as *, P<0.05; ***, P<0.001.
